# Association of biofilm formation and cytotoxic potential with multidrug resistance in clinical isolates of *Pseudomonas aeruginosa*

**Published:** 2019-02-13

**Authors:** Asad Bashir Awan, Juliane Schiebel, Alexander Böhm, Jörg Nitschke, Yasra Sarwar, Peter Schierack, Aamir Ali

**Affiliations:** 1 National Institute for Biotechnology and Genetic Engineering, Faisalabad, Pakistan; 2 Pakistan Institute of Engineering and Applied Sciences, Islamabad, Pakistan; 3 Institute for Biotechnology, Brandenburg University of Technology Cottbus-Senftenberg, Senftenberg, Germany; 4 Institute for Biochemistry and Biology, University of Potsdam, Potsdam, Germany

**Keywords:** Pseudomonas aeruginosa, multidrug resistance, biofilm, cytotoxicity, VideoScan technology

## Abstract

Multidrug resistant (MDR)* Pseudomonas aeruginosa *having strong biofilm potential and virulence factors are a serious threat for hospitalized patients having compromised immunity. In this study, 34 *P. aeruginosa* isolates of human origin (17 MDR and 17 non-MDR clinical isolates) were checked for biofilm formation potential in enriched and minimal media. The biofilms were detected using crystal violet method and a modified software package of the automated VideoScan screening method. Cytotoxic potential of the isolates was also investigated on HepG2, LoVo and T24 cell lines using automated VideoScan technology. Pulse field gel electrophoresis revealed 10 PFGE types in MDR and 8 in non-MDR isolates. Although all isolates showed biofilm formation potential, strong biofilm formation was found more in enriched media than in minimal media. Eight MDR isolates showed strong biofilm potential in both enriched and minimal media by both detection methods. Strong direct correlation between crystal violet and VideoScan methods was observed in identifying strong biofilm forming isolates. High cytotoxic effect was observed by 4 isolates in all cell lines used while 6 other isolates showed high cytotoxic effect on T24 cell line only. Strong association of multidrug resistance was found with biofilm formation as strong biofilms were observed significantly higher in MDR isolates (p-value < 0.05) than non-MDR isolates. No significant association of cytotoxic potential with multidrug resistance or biofilm formation was found (p-value > 0.05). The MDR isolates showing significant cytotoxic effects and strong biofilm formation impose a serious threat for hospitalized patients with weak immune system.

## Introduction

Millions of surgeries are performed annually worldwide but the handling of the postoperative surgical wounds, particularly in developing countries, is inappropriate which often leads to post-surgical infections by opportunistic pathogens (Akenroye et al., 2013[1]; Weiser et al., 2016[43]). *P. aeruginosa* is an ultimate opportunistic gram-negative pathogen which can cause life threatening infections in patients with the compromised immune system. Hence, it is a leading cause of clinical infections all over the world especially in patients admitted in critical care units recovering from post-operative surgical wounds, burns, traumas and pre-exiting lung diseases such as cystic fibrosis. According to Centre for Disease Control more than 51,000 clinical infections are reported each year in the USA with 400 deaths per year (CDC, 2018[5]). European Centre for Disease Prevention and Control (ECDC) has reported 5.8 % prevalence rates of clinical infection in Germany caused by *P. aeruginosa* (Behnke et al., 2017[3]). Individual reports from various regions in developing countries have reported similar incidences but with an alarming increase in drug resistance (Ghane and Azimi, 2014[18]; Nejad et al., 2011[26]; Pathi et al., 2013[28]; Ullah et al., 2016[41]). 

*P. aeruginosa* has a variety of intrinsic, adaptive and acquired resistance strategies against the antimicrobials in use. Synergistic use of these strategies is the basis of multidrug resistance which often leads to failure of therapies in clinical and hospital settings (Fernández and Hancock, 2012[12]). Moreover, the versatile nature of *P. aeruginosa* enables it to survive under drastic nutrient depleted environments due to its ability to use diverse energy sources and attachment to various surfaces. The attachment of motile bacteria to a surface followed by extensive division and entrapping of more motile bacteria leads to the formation of microcolonies. These microcolonies later expand, mature and fuse with each other to form biofilms (Ghanbari et al., 2016[17]). These biofilms decrease the antimicrobial penetration, give protection from host immune system and provide tolerance against antimicrobials by inducing persistence (Mulcahy et al., 2014[25]). In clinical settings, biofilms are formed mostly on indwelling and implanted medical devices used in immunocompromised patients due to improper handling. 

*P. aeruginosa* causes both acute and chronic infections based on their cytotoxic or invasive phenotypes (Fleiszig et al., 1997[15]). Cytotoxic phenotypes induce necrosis within hours of their induction on mammalian cell lines due to strong phospholipase activity (Ramirez et al., 2012[31]). The study of cytotoxicity by pathogenic bacteria in different cell lines is pivotal in understanding bacterial pathogenesis in various body tissues. The cytotoxicity can be determined by differentiating nuclear morphology of the infected and uninfected mammalian cancer cell lines under fluorescence microscopy. DAPI (4′,6′-diamidino-2-phenylindole) is a cell-permeable nucleic acid stain that can be applied to both fixed and unfixed cell lines. Use of DAPI under fluorescence microscopy gives a direct comparison of nuclear to cell morphology (Cummings and Schnellmann, 2004[7]).

We have employed VideoScan technology, which is an automated fluorescence microscopic platform that has been applied for different multiplex assays such as cell pattern recognitions, microbead-based assays (Rodiger et al., 2013[32]), to study biofilm and adhesion assays in clinical isolates of *Escherichia coli *(Frommel et al., 2013[16]; Schiebel et al., 2017[35]). Our objective was to characterize biofilm formation and cytotoxicity of the 34 human clinical isolates of *P. aeruginosa* in correlation with antimicrobial resistance.

## Materials and Methods

### Bacterial isolates

In this study, 34 *P. aeruginosa *isolates (P1-P34) were taken from NIBGE (National Institute for Biotechnology and Genetic Engineering), Pakistan. Out of these, 17 were susceptible to most of the antimicrobials while 17 were multidrug resistant (MDR) i.e., resistant against at least one antibiotic in 3 structurally different antimicrobial groups. These isolates were revived on LB agar supplemented with 1 % glycerol and confirmed their identity by species specific polymerase chain reaction (PCR). The bacterial lysates were prepared by inoculating a single colony in 1 ml of fresh LB broth followed by overnight incubation at 37 °C with 180 rpm shaking. The cultures were centrifuged at 6000 rpm for 5 min, the pellets were dissolved in 300 µl of sterile double distilled water and kept at 99 °C for 10 min. The mixtures were immediately put on ice for 20 min and centrifuged at 6000 rpm for 5 min. The supernatants containing DNA were collected and stored at -20 °C. For PCR, 1 µl of the DNA lysate was added to 25 µl PCR reaction mixture containing *P. aeruginosa *specific primers (Pa-SS-F 5′ GGGGGATCTTCGGACCTCA 3′ and Pa-SS-R 5′ TCCTTAGAGTGCCCACCCG 3′) as described earlier (Spilker et al., 2004[38]).

### Pulse field gel electrophoresis

The PCR confirmed isolates were subjected to pulse field gel electrophoresis (PFGE) using *Bcu*I (*Spe*I) and *Xba*I restriction enzymes (Pournaras et al., 2005[30], Siarkou et al., 2009[37]) with minor modifications to the previously reported method (Hu and Manos, 2015[20]). Overnight cultures (250 µl) were centrifuged and washed twice with 0.9 % NaCl. The bacterial suspension was mixed with 1.2 % PFGE agarose to make gel plugs. These plugs were digested overnight with proteinase K. The plugs were washed thrice with 1X TE buffer and digested with respective restriction enzyme. The plugs were loaded in 1.2 % PFGE agarose gel along with molecular marker (Lambda ladder PFG, New England Biolabs). The gel was run in 0.5X TBE buffer containing 100 µmol/L thiourea using CHEF DR-III variable angle system (Bio-Rad). The equipment was set as angle 120°, voltage 6V, pulse of 5-50, duration 22 h. Then the gel was immersed in ethidium bromide (0.5 µg/ml) for 15 min and then visualized by gel doc system. The isolates having three or more different bands were considered as different PFGE type. 

### Biofilm formation assays 

The overnight LB broth cultures of *P. aeruginosa *were brought to OD_600_ = 1 and diluted (1:100) with four different media (two enriched media: BHI broth and LB broth, and two minimal media: M9 with 0.2 % glucose and M9 with 0.2 % glycerol). The 200 µl of the bacterial suspension was allowed to make biofilm in each well of the 96 well flat bottom polystyrene plates (Greiner Bio-One GmbH, Frickenhausen, Germany). *E. coli* strain K-12 MG1655 F'tet Δ*traD* was used as biofilm forming positive control. The plates were covered with sealing films and incubated overnight at 37 °C for biofilm formation. Non-adherent bacteria from the wells were aspirated and attached biofilms were washed once with 200 µl of sterile 0.9 % NaCl. The biofilm formation potential of the 34 isolates in each media was tested in triplicate with three independent experiments in each method. After this procedure, two independent batches were subjected to two different detection methods while the batch after completion of VideoScan detection method was further subjected to crystal violet staining.

#### Crystal violet (CV) detection method

For CV staining, a 200 µl volume of 0.1 % CV was added in each well and incubated at room temperature for 10 min. The plates were washed twice with 200 µl of sterile 0.9 % NaCl solution. Then 200 µl of 95 % ethanol was added to each well and kept for 10 min to extract surface bound CV. The solution from these wells was aspirated and transferred to another blank 96 well plate. The OD at 570 nm was measured with a microplate reader (Sunrise; Tecan GmbH, Germany). All observations were analyzed according to mean + 3 standard deviations of negative control for each plate (Stepanovic et al., 2000[39]).

#### VideoScan (VS) detection method

An indigenously optimized automated method (Schiebel et al., 2017[35]) with minor modifications was applied for visual detection of biofilm formation. For biofilm staining, 5 µM solution of SYTO 9 in 0.9 % NaCl was added to each well and the plates were placed in the dark at room temperature for 10 min. The wells were then washed once with 200 µl of 0.9 % NaCl. The wells were filled with 0.9 % NaCl and proceeded for automated VideoScan analysis. Reference microbeads (PolyAn GmbH, Berlin, Germany) were used as internal standard and the median intensity of fluorescence of these beads was used to calculate relative fluorescence intensity (relFl) of each well (Schiebel et al., 2017[35]). The intensity of wells containing only 0.9 % NaCl was used as negative control for each of the plates. The overall well fluorescence was measured using automated VideoScan technology. The fluorescence detection software package was modified from previously reported 'FastFluoScan' (Schiebel et al., 2017[35]) to 'Globalwellintensity' which measured intensity from the whole well instead of central 4 mm x 4 mm square. All values were analyzed using cut-off values based on relFl_c_ (mean relFl + 3SD of blank wells) and the isolates having relFl below relFl_c_ were categorized as non-biofilm forming. Whereas biofilm forming isolates were categorized as weak (relFl = relFl_c_ to 2x relFl_c_), moderate (relFl = 2x relFl_c_ to 3x relFl_c_) and strong (relFl > 3x relFl_c_). 

#### VideoScan cytotoxicity assay

The infection of *P. aeruginosa* on epithelial cells ultimately leads to loss of cell membrane integrity and release of cytoplasmic contents which finally results in cell detachment that overall is known as cytotoxicity (Bucior et al., 2014[4]). Previously DAPI has been widely used in different fluorescence based cytotoxicity assays (Cummings and Schnellmann, 2004[7]). We allowed the bacterial isolates to infect different cell lines for 3 hrs and the monolayer confluences' with and without bacteria were compared using automated imaging of 96-well plates by Video-Scan technology. The remaining cells in the wells were visualized with nuclear staining (DAPI) (Ude et al., 2017[40]) and detected as a retained monolayer. The disruption of mammalian cell monolayers was interpreted as directly proportional to the bacterial cytotoxicity. 

Three cell lines were used viz., HepG2 (human liver cells), LoVo (human colon cells) and T24 (human urinary bladder cells). The cell monolayers were prepared in 96-well plates (Nunclon, ThermoFisher) using DMEM/Ham's F12 medium (Millipore) supplemented with 10 % fetal bovine serum (Millipore), 2 mM L-glutamine and 100 IU/100 µg per ml penicillin/streptomycin (Millipore). The cell line plates with more than 90 % confluency were washed with 1X PBS and 100 µl of fresh media with 10 % FBS (without any antibiotics) was added to each well. Overnight growth (OD 1 at 600 nm) of each *P. aeruginosa* isolate was added in three wells after calculating their dilutions in fresh media to ensure multiplicity of infection (MOI) as 100 bacteria by eukaryotic cell (Schierack et al., 2013[36]). The cell line plates were then incubated at 37 °C with 5 % CO_2_ for 3 hrs. After incubation, the plates were washed with 100 µl of 1X PBS. The plates were fixed with 4 % paraformaldehyde solution in 1X PBS for 1 hr at 4 °C. The plates were washed thrice with 1X PBS followed by the addition of 100 µl of blocking buffer (1X PBS containing 0.5 % BSA) for 5 min at room temperature. The blocking buffer was aspirated and 50 µl of DAPI staining (50 μg/ml) solution was added for 30 seconds at room temperature. After washing the cell lines twice with 1X PBS, a 100 µl of 1X PBS was added to each well and the plates were processed for automated imaging using VideoScan technology. The experiments were performed in triplicates and in 5 independent batches for each cell line. 

#### Statistical analysis

For statistical analysis, ANOVA was used to validate cut-off values for 'Strong', 'Moderate' and 'Weak' biofilm formation obtained by VideoScan method. Pearson values were used to find correlation when biofilms were compared based on different media or detection methods and also to correlate cytotoxic effects of the isolates on three different cell lines. The correlation was categorized as strong (Pearson value > 0.7), moderate (Pearson value 0.3 to 0.7) and weak (Pearson value < 0.3). Chi-square tests were used to check the significant differences between MDR or non-MDR isolates based on their biofilm formations and cytotoxic effects.

## Results

### Bacterial isolates and PFGE

**Figure 1:**
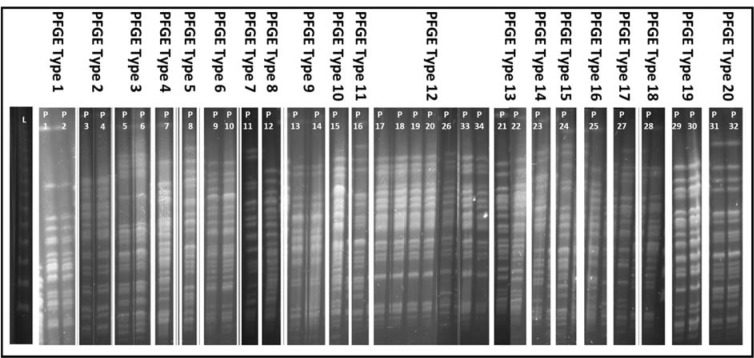
PFGE types of *P. aeruginosa* isolates based on SpeI restriction enzyme

All 34 isolates were successfully revived and their identification was confirmed by PCR amplification of 956 base pairs fragment of 16S rRNA gene specific for *P. aeruginosa*. PFGE grouped 17 MDR isolates into ten PFGE types and 17 non-MDR isolates into eight PFGE types while two PFGE types contained one MDR as well as one non-MDR isolate each. Restriction with either *Spe*I or *Xba*I revealed same PFGE types except for one isolate (P12) which was grouped in a separate PFGE type by *Spe*I (Figure 1(Fig. 1)).

### Biofilm formation assay

#### CV detection method

The biofilm formation by the isolates when compared within two enriched or two minimal media was found strongly and positively correlated (Pearson value = 0.92) whereas, a moderate correlation was found between enriched and minimal media (Pearson value = 0.48). Out of 34, 8 (23.5 %) isolates showed strong biofilm formation in all four media, among them seven were MDR while one was non-MDR. While comparing different media, nine isolates (four MDR and five non-MDR) showed strong biofilm formation only in enriched medium (BHI and LB media) and two isolates (one MDR and one non-MDR) showed this potential only in M9 minimal media. 

#### VS detection method

**Figure 2:**
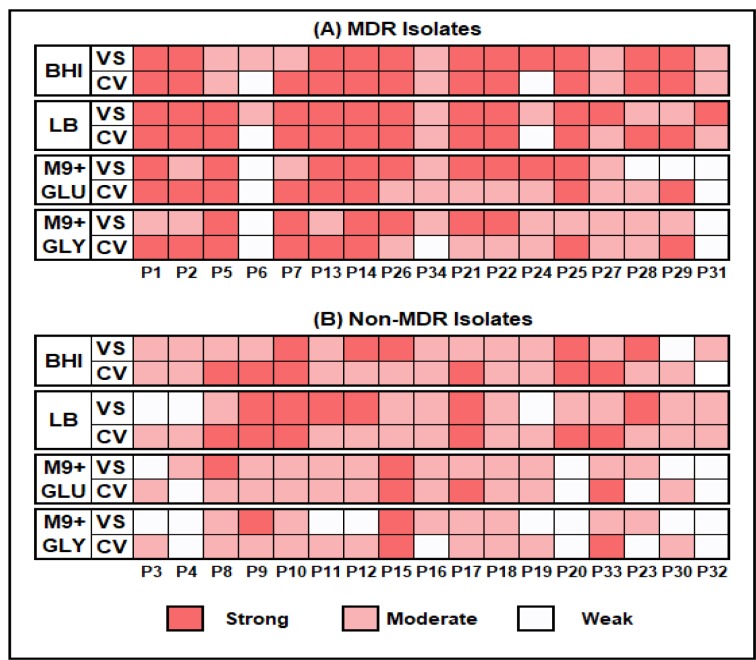
Biofilm formation potential by MDR and Non-MDR *P. aeruginosa* isolates in different media by CV (Crystal Violet) and VS (VideoScan) detection methods. BHI = Brain Heart Infusion broth, LB = Luria Bertani broth, M9+GLU = M9 minimal medium + 0.2 % glucose and M9+GLY= M9 minimal medium + 0.2 % glycerol

**Figure 3:**
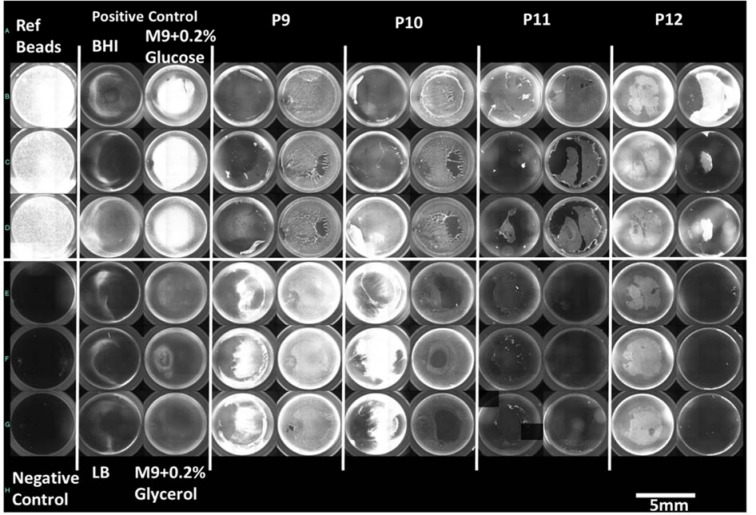
Overview image of the biofilms formed by *P. aeruginosa* detected by VideoScan method

Four MDR isolates showed strong biofilm formation in all four media while no non-MDR isolate showed such potential. However, five isolates (four MDR and one non-MDR) showed strong biofilm formation in any three media and 1 non-MDR isolate showed strong biofilm in minimal media only. A moderate correlation was found among all four media [Pearson value ranging from 0.3 (between LB and M9 with glucose) to 0.65 (between two minimal media)]. Figure 2(Fig. 2) shows biofilm formation potential of MDR and non-MDR isolates in different media as detected by three methods whereas, Figure 3(Fig. 3) shows a compiled visual image of formed biofilm in a 96 well polystyrene plate.

#### Crystal violet after SYTO 9 (CVaS9)

The correlation among biofilms formed in enriched media was found moderate (Pearson value=0.59) while in minimal media it was strong and positive (Pearson value= 0.72). One MDR and one non-MDR isolate showed strong biofilm in all four media. Four isolates (three MDR and one non-MDR) showed strong biofilm formation in at least three media.

#### VideoScan cytotoxicity assay

**Figure 4:**
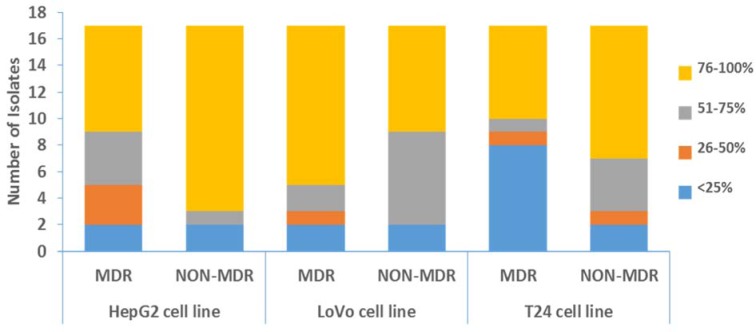
Distribution of *P. aeruginosa* isolates resulted in varying percentages of retained cell monolayers

**Figure 5:**
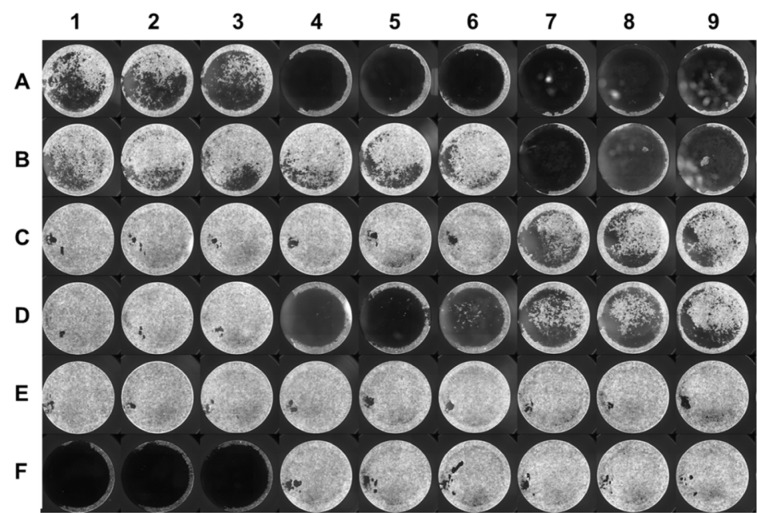
VideoScan image of a T24 cell line plate after completion of cytotoxicity assay. Different isolates were added in triplicates (row-wise) on the 96-well plate with intact monolayers, whereas wells E7-E9 and F7-F9 were negative controls (without bacteria)

The bacteria infected the cell lines and resulted in cytotoxic detachment of monolayers from the well surfaces. There was no significant difference (p-value > 0.05) observed in cytotoxicity of MDR and non-MDR isolates. The isolates caused more monolayer disruption in case of T24 cell lines as compared to other two cell lines. The distribution of MDR and non-MDR isolates resulted in varying percentages of retained monolayers is shown in Figure 4(Fig. 4). Four isolates (two MDR and two non-MDR) showed high cytotoxicity (retained monolayer < 25 %) in all three cell lines. Whereas, six other isolates (five MDR and one non-MDR) showed high cytotoxicity (retained monolayer < 25 %) in T24 cell line only. Figure 5(Fig. 5) shows the combined captured image of a 96 well plate after infection assay as detected by VideoScan technology. 

## Discussion

*P. aeruginosa* is among the most critical and resistant bacteria according to World Health Organization for which new antimicrobials are urgently required (WHO, 2017[44]). The multidrug resistance among *P. aeruginosa* isolates was found 13 % and 10.3 % in USA and Europe respectively (CDC, 2018[5]; ECDC, 2017[9]). Whereas, the multidrug resistance in developing countries is high due to multiple factors. In Pakistan, occurrence of multidrug resistance has been reported earlier as 16 % (Ullah et al., 2016[41]), 30 % (Khan et al., 2014[21]), 36.5 % (Mansoor et al., 2015[23]), 39.4 % (Samad et al., 2017[34]) and 63.2 % (Ali et al., 2015[2]). Multidrug resistance reported from India (47.7 %) and Iran (54.5 %) also highlighted its severity (Gill et al., 2016[19]; Saderi and Owlia, 2015[33]). 

Due to potent biofilm formation and metabolically versatile nature along with innate and acquired resistance, *P. aeruginosa* is classified as one of the ESKAPE pathogens (*Enterococcus faecium, Staphylococcus aureus, Klebsiella pneumoniae, Acinetobacter baumannii, P. aeruginosa *and* Enterobacter spp.*) which are capable of escaping bacteriocidal activity of different antimicrobial and antiseptic groups (Pendleton et al., 2013[29]). We studied biofilm formation in enriched and minimal media to understand the potential of *P. aeruginosa *to adapt in nutrient depleted environments. Although the potential of biofilm formation varied with different media and detection methods, we found significantly higher potential of strong biofilm formation in enriched media as compared to minimal media. Moreover, MDR isolates showed strong biofilm formation as compared to non-MDR isolates which is in line with the previous reports where majority of MDR *P. aeruginosa* isolates showed stronger biofilm formation (El Galil et al., 2013[10]; Elhabibi and Ramzy, 2017[11]; Ghanbari et al., 2016[17]; Lima et al., 2018[22]). Similarly, a study from Iran reported higher prevalence of MDR isolates who have slightly stronger biofilm production in the enriched medium than non-MDR isolates (Corehtash et al., 2015[6]) whereas, carbapenem resistant *P. aeruginosa *strains also showed more biofilm potential than carbapenem susceptible isolates (Ochoa et al., 2013[27]). 

We employed two different methods to quantify the formed biofilms viz., using crystal violet and VideoScan. The VideoScan method, in addition to detecting biofilm intensities, also captures images of the formed biofilms to understand their textures. The formed images can also detect the disruption of biofilm at a particular position in the well due to pipetting and resulted in lower fluorescent intensity that can mislead as weak biofilm. Although the number of isolates showing strong biofilms in 3 or more media was found similar by both crystal violet (10/34) and VideoScan (9/34) methods, the number of isolates detected as 'strong biofilms producers' in all media varied in case of both methods. The overall comparison of crystal violet and VideoScan methods showed moderate correlation (Pearson value = 0.4 to 0.5). This might be due to the biofilms formed by *P. aeruginosa* isolates at meniscus level (solid-liquid-air interphase) which the VideoScan method was not able to quantify (Schiebel et al., 2017[35]). Increasing the volume of SYTO 9 staining solution in the well to completely immerse the meniscus phase might result in a strong positive correlation between VideoScan and crystal violet methods. After detection of biofilms by the VideoScan method, we subjected the same 96-well biofilm plates to crystal violet staining (CVaS9) assuming that SYTO 9 staining will not affect the crystal violet staining. The overall biofilm quantifications by CV and CVaS9 methods showed strong positive correlation (Pearson value= >0.7). However, the decrease in a number of isolates showing strong biofilm formation in 3 or more media by CVaS9 method (6/34) might be due to repeated pipetting in previously used (VS) plates. 

Although an intact tissue is relatively resistant to cytotoxic effects of *P. aeruginosa*, however an injured or non-healthy tissue might get infected easily (Fleiszig et al., 1997[14]). During infection, *P. aeruginosa* isolates multiply and induce their type III secretion system (TTSS) which results in the direct release of toxins into the host cells. These toxins are responsible for rapid cytotoxicity and necrosis of the host cells and hence are helpful in evading from host defenses (Filopon et al., 2006[13]). In our study, we have used DAPI staining to detect retained monolayer after infection as the free nucleic acid from the destroyed cells which was believed to be removed during washing steps in the procedure. However, complementation with propidium iodide (PI) staining can differentiate live and dead cells in the retained monolayer. After 3 hrs of infection, the retained monolayer ranged from 14 % to 95 % in HepG2 cell lines and 5 % to 95 % in both LoVo and T24 cell lines. These results were similar to a study where pyocyanin (a secretory product of *P. aeruginosa*) inhibited 7 % to 84 % growth of HepG2 cell line due to its cytotoxic effect (Mohammed et al., 2014[24]). The variation among cytotoxic effects may be due to variability in inducing TTSS and in the production of various toxins (Vázquez-Rivera et al., 2015[42]) that also varies among different cell lines (Dasgupta et al., 2015[8]). 

In summary, the MDR isolates of *P. aeruginosa* showed stronger biofilm forming potential than non-MDR isolates and stronger biofilms were observed in enriched media as compared to minimal media. No significant association was found between antimicrobial resistance and cytotoxic effect (p > 0.05) and no significant difference was found when cytotoxic effects were compared among strong, moderate and weak biofilm forming isolates (p > 0.05). However, more than six MDR isolates were found showing strong biofilm formation and higher cytotoxic effects depicting a lethal combination of bacterial armory that poses a serious concern for public health.

## Acknowledgements

This study was sponsored by Indigenous PhD Fellowship Program of Higher Education Commission (HEC), Pakistan and Federal Ministry of Education and Research, Germany (BMBF InnoProfile-Transfer 03IPT611X). 

## Disclosure

The authors declare that they have no conflict of interest.
